# Supplementary figures and images for: Oxytocinergic projection from the hypothalamus to supramammillary nucleus drives recognition memory in mice
Source: PLoS One. 2023 Nov 16;18(11):e0294113. doi: 10.1371/journal.pone.0294113 (PMC10653413; doi:10.1371/journal.pone.0294113)

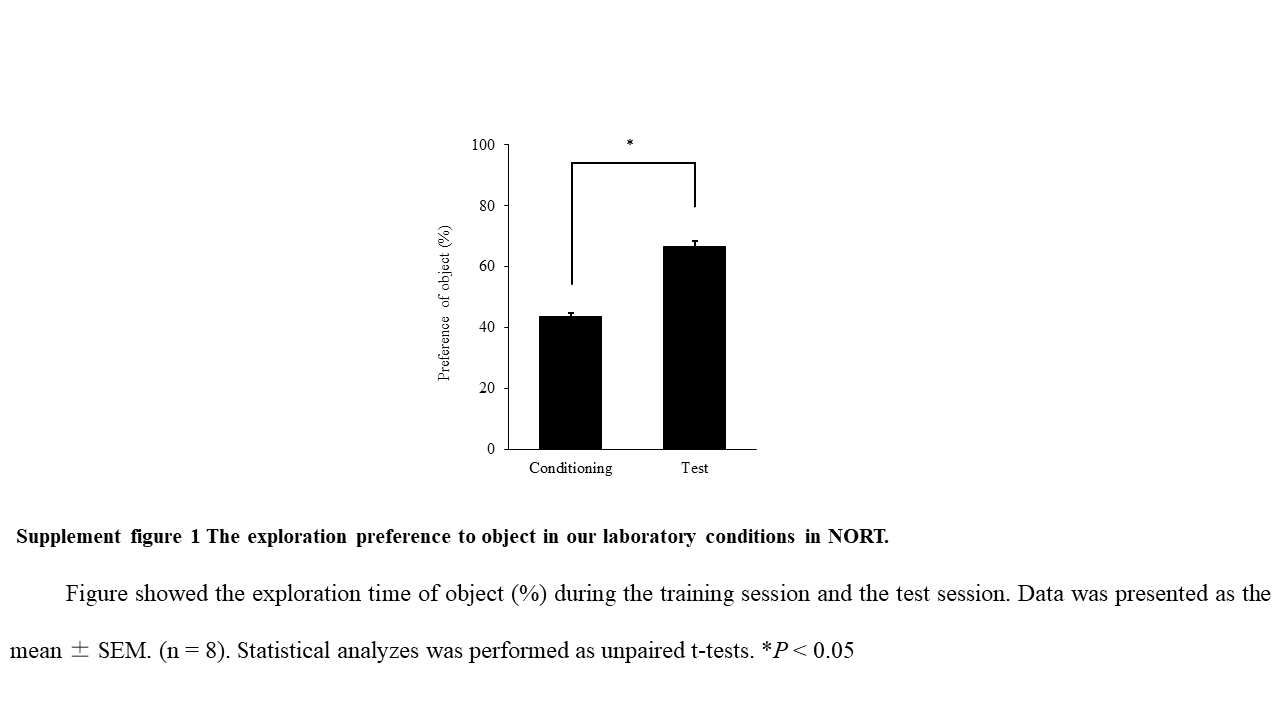

Supplement: S1 Fig — Figure showed the exploration time of object (%) during the training session and the test session. Data was presented as the mean ± SEM. (n = 8). Statistical analyzes was performed as unpaired t-tests. *P < 0.05. (TIF) [file pone.0294113.s001.tif]

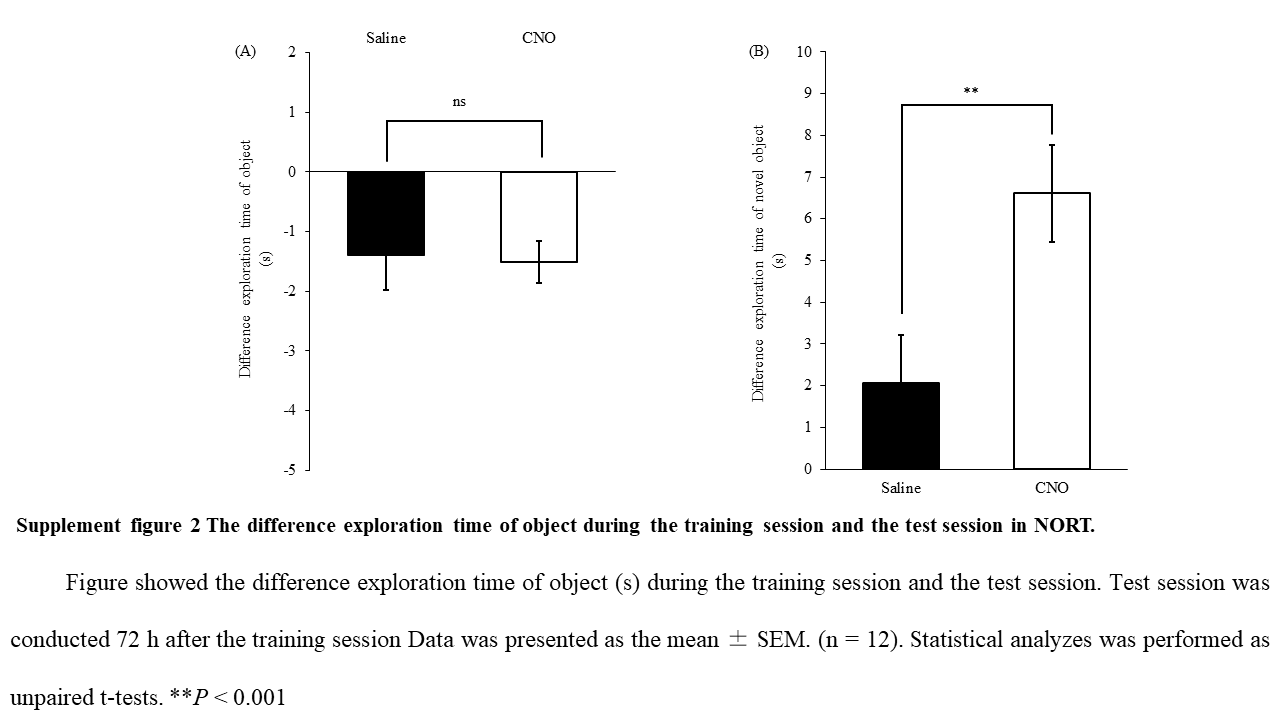

Supplement: S2 Fig — Figure showed the difference exploration time of object (s) during the training session and the test session. Test session was conducted 72 h after the training session Data was presented as the mean ± SEM. (n = 12). Statistical analyzes was performed as unpaired t-tests. **P < 0.001. (TIF) [file pone.0294113.s002.tif]

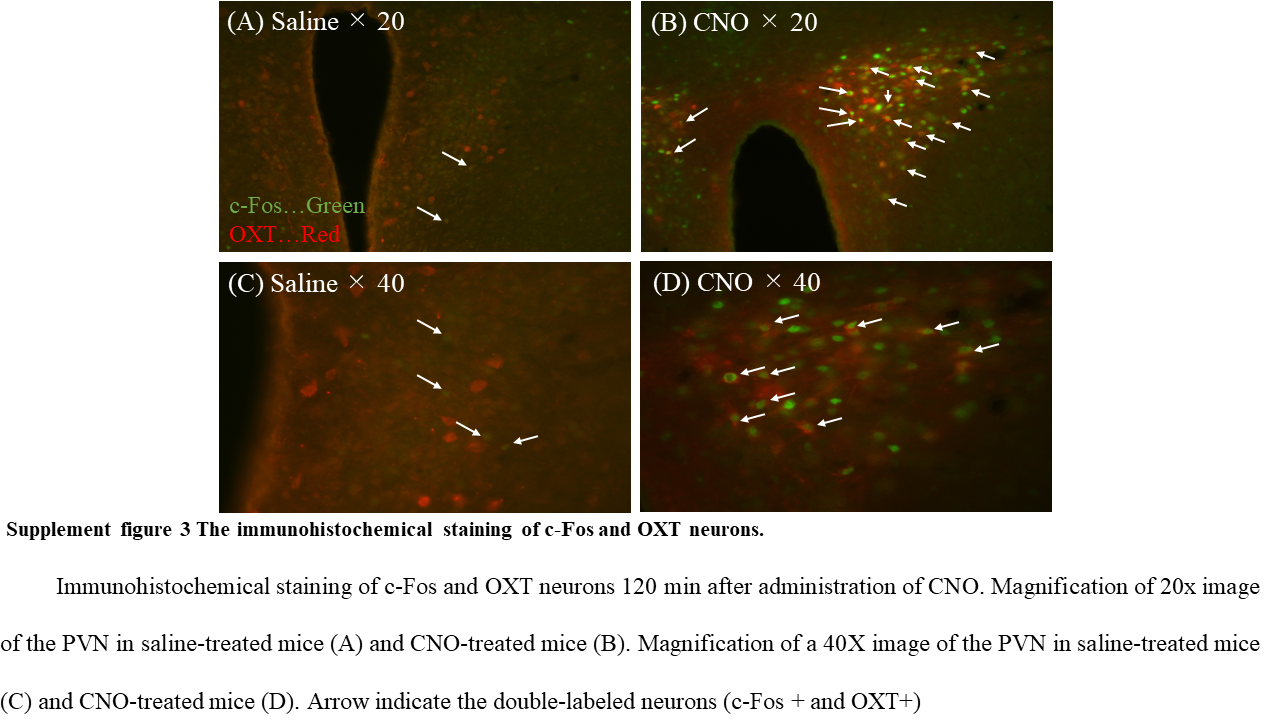

Supplement: S3 Fig — Immunohistochemical staining of c-Fos and OXT neurons 120 min after administration of CNO. Magnification of 20x image of the PVN in saline-treated mice (A) and CNO-treated mice (B). Magnification of a 40X image of the PVN in saline-treated mice (C) and CNO-treated mice (D). Arrow indicate the double-labeled neurons (c-Fos + and OXT+). (TIF) [file pone.0294113.s003.tif]
